# Atlantic Canadians’ Sensory Perception of Couscous Made with Sugar Kelp (*Saccharina latissma*)

**DOI:** 10.3390/foods13182912

**Published:** 2024-09-14

**Authors:** Mackenzie Gorman, Laura Baxter, Rachael Moss, Matthew B. McSweeney

**Affiliations:** School of Nutrition and Dietetics, Acadia University, Wolfville, NS B4P 2K5, Canada; 153760g@acadiau.ca (M.G.); 160338b@acadiau.ca (L.B.); 145961m@acadiau.ca (R.M.)

**Keywords:** seaweed, macroalgae, nutritional benefits, sensory perception, food product development, sustainable ingredients, check all that apply, consumer perception

## Abstract

Sugar kelp (*Saccharina latissma)* has many nutritional benefits and has been identified as a rich source of fibre, vitamins, and antioxidants. However, it is not regularly consumed in the Western world, and the sensory perception of foods containing sugar kelp must be investigated to increase acceptance in North America. This study evaluated consumers’ (n = 99) sensory perception of couscous with increasing amounts of sugar kelp (0% (control), 4%, 6%, 8%, and 10% wt/wt). Furthermore, consumers’ purchase intent, liking, and emotional response to couscous with added sugar kelp was evaluated with and without nutritional information. Sugar kelp at 6% incorporation did not impact the consumers’ liking scores (“Like Slightly” on the hedonic scale), but at 8% the consumers’ liking significantly decreased (“Neither Like nor Dislike”). The 8% and 10% levels of sugar kelp addition led to astringency, bitter, hard, brackish, fishy, and chewy attributes being perceived by the consumers. The consumers identified they preferred samples that had soft, savoury, salty, and bland flavours and disliked samples that were brackish and gritty. The nutritional information did not increase overall liking scores, purchase intent, or emotional response. However, the inclusion of sugar kelp in the couscous did lead to an increased selection of positive emotions like happy, joyful, pleasant, and enthusiastic. Overall, the consumers were interested in foods containing seaweed and believed they were nutritious. The results indicated that sugar kelp could be added to couscous up to 6% wt/wt without impacting overall liking.

## 1. Introduction

Over the last decade, aquaculture and the global seaweed trade have grown rapidly. From 2005 to 2015, global seaweed production increased from 13.5 to 30.4 million tons per year, deeming it one of the fastest-growing food sectors around the world [1]. More than 99% of seaweed produced is from Asian countries. However, there has been a large market expansion in Western and European countries due to the rising popularity of seaweed-based products [2,3], and studies have been conducted on increasing production of seaweed in North America [4,5]. The global market for algae was identified to be EUR 594 million in 2018, and it has been projected to reach EUR 1131 million by 2027 [6]. Seaweed’s applications extend beyond consumption for human health benefits and is a sought-after product due to its potential to be a new protein source, a component in fertilizers and renewable biofuels, an ingredient in nutraceuticals, medicine, and cosmetics, and a food additive [7]. Furthermore, seaweed production has numerous environmental benefits, making it a highly sustainable farming process due to the lack of land, fertilizers, pesticides, and freshwater resources required for cultivation [8]. Beyond sustainable aquaculture purposes, seaweed offers potential solutions to other environmental challenges because it protects vulnerable ecosystems from eutrophication, aids in carbon/nitrogen sequestration, and naturally improves the water quality necessary for healthy ecosystem maintenance and growth [3,9]. One of the most promising and abundant seaweed species for cultivation is *Saccharina latissma*, also commonly known as “sugar kelp”.

*Saccharina latissma* is a brown marine algae that belongs to the Laminariaceae family (kelp) [10]. Sugar kelp is a cold-water seaweed species that thrives in shallow seas and sheltered shorelines around coastal regions worldwide [11]. It is commonly cultivated around coastlines in Northern Europe, Japan, China, and along the eastern and western coastlines of North America, such as in Alaska, Maine, and Nova Scotia. Sugar kelp grows by attaching itself to rocky seabeds, allowing it to flourish in depths up to 30 m [12]. It exhibits impressive growth rates during colder months and grows fastest from winter to early spring. With its huge growth potential, sugar kelp can grow up to 5 m and 20 cm wide over a lifespan of two to four years [13]. The optimal growing temperature for sugar kelp is between 5 and 17 °C, while certain varieties can tolerate colder temperatures [14].

*Saccharina latissma* earns its common name, “sugar kelp”, from its drying process. When sugar kelp is left to dry, a white, sweet powder called mannitol forms on its fronds. Mannitol is a naturally occurring sugar alcohol and can be easily and cheaply isolated to be used as a sugar alternative, bulking agent, and laxative, as well as in biofuel production and clinical applications [15]. Sugar kelp is described as having a strong, salty, savoury, and umami-rich flavour [16]. It is enjoyed as a sea vegetable, a food ingredient, and as a seasoning agent to add depth to soup and sauce bases [2]. The reasons for the consumption of sugar kelp extend beyond its varied sensory profile; it is also a functional food with a high nutrient density.

Sugar kelp is a rich source of fibre, vitamins, minerals, and antioxidants. Sugar kelp’s mineral content has been identified to be among the highest in comparison to other seaweed variants and even higher than some terrestrial plants, meaning there has been growing interest in its health benefits in the research fields of agriculture and nutrition [17]. The literature has shown that sugar kelp exhibits antioxidant and immunomodulatory effects, which can help the body’s immune system fight infection and diseases [18]. Furthermore, sugar kelp is inherently high in iodine and iron, which is crucial for healthy thyroid function. It is also a valuable nutritional source of iron, zinc, titanium, vitamin C, and vitamin K, which all play a role in the prevention of non-communicable diseases [17]. Sugar kelp also contains an elevated quantity of polysaccharides that demonstrate an anti-obesity, anti-diabetic, anti-inflammatory, antioxidant, and hepatoprotective effect in the body [18]. The polysaccharides specifically found in sugar kelp can even function as a prebiotic, aiding to support a healthy gut function by promoting the growth of beneficial bacteria within the gut [18].

Emerging research suggests that the polysaccharides in brown seaweed and specifically sugar kelp hold the potential to aid in weight management due to their anti-obesity and anti-adipogenic effects [15]. Kim and colleagues [18] discovered a link between the consumption of sugar kelp and a drastic reduction in body weight in obese mice. This was ultimately attributed to how sugar kelp consumption increased metabolic rates, energy expenditure, physical activity, and reduced adipose tissue inflammation, which can suppress the development of insulin resistance. The elevated metabolic rate was in part due to sugar kelp’s high fibre content, which can be fermented by the gut bacteria to increase energy expenditure. This finding suggests that sugar kelp can alter the gut microbiota composition positively to help aid in weight reduction. The research on sugar kelp’s nutritional benefits is still limited in scope and ongoing; however, the emerging research suggests it is an appealing choice to consumers due to its multitude of health advantages and nutritional benefits.

Limited studies have characterized the sensory attributes and consumer acceptance of sugar kelp or products that contain it as an ingredient. Most of the sensory evaluation studies on sugar kelp have focused on fermentation, blanching, and freezing. For example, Bruhn and colleagues [2] conducted a Quantitative Descriptive Analysis panel to develop a lexicon of flavour descriptors and attributes to describe fresh versus heat-treated and fermented sugar kelp. The study finalized 11 key attributes to describe sugar kelp, those being “slimy”, “yellow-green”, “smell of sea”, “iron”, “boiled green vegetables”, “bite”, “sour”, “sweet”, “salty”, “bitter”, and “umami”. The fermentation process caused a significant reduction in the “sea smell” and reduced the slimy visual appearance. However, it also reduced the salty and umami taste of sugar kelp. Additionally, Akomea-Frempong and colleagues [9] conducted a study to explore how different preservation processes affect the sensory attributes and consumer acceptance of sugar kelp. Sauerkraut made from fermented sugar kelp received a higher hedonic score than its raw counterpart. This was attributed to the increased saltiness; however, consumers expressed dissatisfaction with the fishy and pungent flavour that the sugar kelp added to the sauerkraut.

Further exploration into the integration of sugar kelp into food products must be carried out to gain consumer acceptance in North America. Couscous was chosen for this study as it is a cereal grain-based product and is considered a savoury dish [19]. It was identified in a past study that Atlantic Canadians are interested in the addition of seaweed to cereal grain-based products and savoury products [20]. Also, a past study identified that *Chlorella vulgaris* could successfully be added to couscous [21]. As such, the study evaluated the addition of sugar kelp to couscous using hedonic scales and check-all-that-apply (CATA) questions. Furthermore, the study investigated how nutritional information impacts the consumer’s perception of sugar kelp added to couscous.

## 2. Materials and Methods

### 2.1. Samples

Couscous (durum wheat semolina; Loblaws Inc., Toronto, ON, Canada) was purchased from a local grocery store (batch numbers were matched for consistency). Sugar kelp (Maine Coast Sea Vegetables; Hancock, ME, USA) was purchased and then ground and passed through a 60-mesh sieve for consistency. Couscous was mixed with the ground sugar kelp at different levels: control (0% sugar kelp), 4% (wt/wt) sugar kelp (referred to as 4SK), 6% sugar kelp (6SK), 8% sugar kelp (8SK), and 10% sugar kelp (10SK). The levels chosen to be included in this study were based on preliminary evaluations by research assistants working in the sensory lab. Furthermore, a previous study identified another seaweed variety, *Chlorella vulgaris*, that could be added at 6% wt/wt, and therefore, we wanted to build on this study by increasing the amount of sugar kelp added to the couscous [21]. The couscous (57.09 g) without sugar kelp (control) and the couscous with added ground sugar kelp were added to boiling water (156 g) and then removed from the heat and allowed to sit for 10 min undisturbed [22]. The couscous was then agitated (or fluffed) with a fork. All the couscous products were measured into 100 g portions, and 13.5 g of olive oil and 2.5 g of salt were added; then, the couscous was fluffed again with a fork [22].

### 2.2. Participants

Consumers (n = 99, 54 females, 45 males, average age of 33.3 ± 9.9) who self-identified as having consumed couscous in the last two months were recruited. Consumers were screened for allergies or sensitivities to the ingredients. All participants received information about the study and gave informed consent before participating in it. The first trial included 99 consumers, and then they were invited to participate in the follow-up study two weeks later. A total of 90 consumers participated in the second trial.

### 2.3. Sensory Procedure

Both sensory trials took place in the Centre for the Sensory Research of Food (Acadia University, Wolfville, NS, Canada). The questionnaire was completed using a paper questionnaire in individual sensory booths in a temperature-controlled room.

The procedure was adapted from Gorman and colleagues [23]. The consumers (n = 99) evaluated the five different couscous samples (control, 4SK, 6SK, 8SK, 10SK) one at a time, and the samples were presented following a completely randomized design. The participants also received a glass of distilled water and slices of McIntosh apples as palate cleansers. There was a minute break between samples. The consumers evaluated the samples using a nine-point hedonic scale (1 = Dislike Extremely and 9 = Like Extremely) for overall liking, as well as their liking of appearance, flavour, and texture. They also answered a CATA question including different sensory attributes (soft, hard, gritty, moist, herbal, salty, fishy, sweet, bitter, astringent, bland, nutty, grainy, smooth, chewy, sour, umami (savoury), aftertaste, metallic, fatty, tough, crunchy, brackish, and musty). Attributes were included based on evaluations by research assistants employed in the sensory lab and past studies investigating seaweed and couscous [9,22,23,24,25,26,27]. CATA attributes were randomized across participants and samples [28]. Consumers were instructed to select all of the attributes they perceived in the sample. Consumers also had the option to provide additional comments about the samples, as well as complete questions about seaweed (based on the study by Lamont and McSweeney [29]) and demographic questions.

The consumers were then invited to return to the sensory lab for a second trial, and 90 consumers returned. The participants received three samples: one control, 6SK (based on the results of the previous sensory trial), and 6SK with nutritional information about sugar kelp. The participants received the blinded samples first (in a randomized order), and then they received the 6SK sample with nutritional information. The nutritional information stated, “This sample contains sugar kelp. Sugar kelp has been found to be a rich source of fibre, antioxidants, minerals, and vitamins including vitamin C, vitamin K, iron, calcium, iodine, and magnesium” [30]. Similar to the above, the participants also received distilled water and apple slices to cleanse their palates. There was a minute break between samples. The participants evaluated the samples using hedonic scales for their overall liking (1 = “Dislike Extremely” and 9 = “Like Extremely”) and their purchase intent (a five-point scale from 1 = “Definitely would not purchase” to 5 = “Definitely would purchase”) [31]. The consumers also identified their emotional response using the CATA format of the EsSense25 profile [32] and were asked, “How does this sample make you feel? Select all that apply.” [33]. They also answered demographic questions.

### 2.4. Statistical Analysis

The results were transferred from the paper questionnaire to spreadsheets in Microsoft Excel. The results from the hedonic scales from both sensory trials and the purchase intent scale in the second trial were evaluated using a two-way Analysis of Variance (ANOVA), and when a significant difference existed, a Tukey’s Honestly Significant Difference test was conducted. The results of the CATA questions (first trial = sensory properties, second trial = emotions) were evaluated following the procedure by Meyners and colleagues [34] using a Cochran’s Q test (followed by pairwise comparisons using Sheskin’s critical differences (Supplementary Table S1)) and correspondence analysis. Furthermore, a penalty lift analysis (adapted from Meyners and colleagues [34]) was conducted using the results of the sensory property CATA question in the first sensory trial and the overall liking scores. The responses to the EsSense25 CATA were statistically analyzed using McNemar’s test with Bonferroni correction. A two-sided *t*-test compared the mean hedonic scores for the control and sample 6SK from the first trial (n = 99) and the second trial (n = 90). Descriptive statistics were used to evaluate the demographic questions and the seaweed questions. All analyses were completed in XLSTAT (Lumivero, Denver, CO, USA) in Microsoft Excel Version 16.87.

## 3. Results

### 3.1. Sensory Trial #1—Different Levels of Sugar Kelp Incorporation

The mean liking scores are outlined in Table 1. The addition of sugar kelp significantly increased the consumers’ liking of the appearance of samples 4SK, 6SK, and 8SK (“Like Slightly” on the hedonic scale) in comparison to the control (*p* < 0.05; “Neither Like nor Dislike”). The consumers’ liking of the flavour and their overall liking of the 4SK and 6SK samples were not significantly different from the control (“Like Slightly” on the nine-point hedonic scale; *p* < 0.05). The scores (flavour and overall liking) for 8SK and 10SK were significantly lower than those of the control and the 4SK and 6SK samples (“Neither Like nor Dislike”; *p* < 0.05). The consumers scored the texture of the control (“Like Slightly”) significantly higher than all other samples (*p* < 0.05).

**Figure 1:**
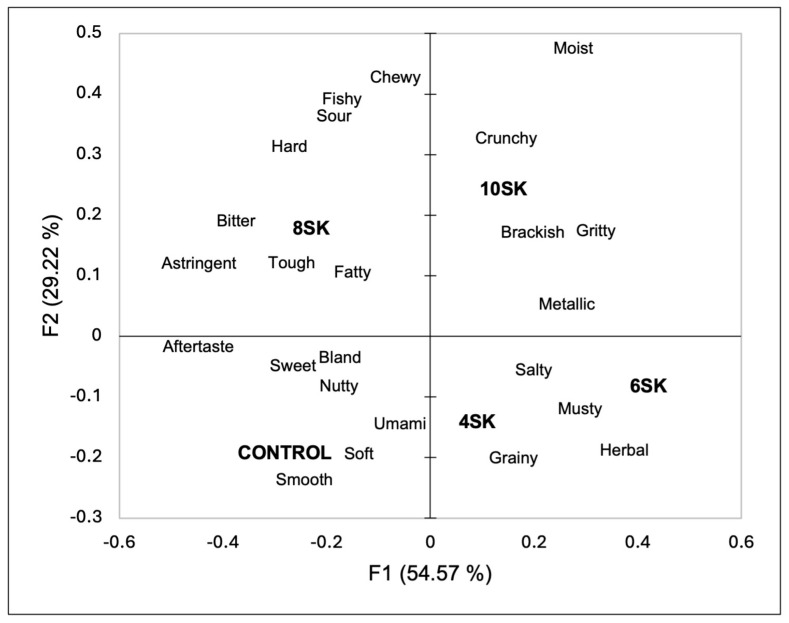
Biplot based on the first two dimensions of the correspondence analysis for the samples and the sensory properties included in the CATA question.

The consumers identified their sensory perception using a CATA question (Figure 1 and Supplementary Table S1). The first two dimensions of the correspondence analysis explained 83.79% of the variation (54.57% on the first dimension and 29.22% on the second dimension). The 8SK and 10SK samples were separated from the other samples by the second dimension and, in turn, were separated from each other by the first dimension. The 8SK sample was associated with astringent, tough, bitter, fatty, hard, sour, fishy, and chewy. While the 10SK was perceived to be crunchy, brackish, metallic, gritty, and aftertaste. Samples 4SK and 6SK were grouped and associated with salty, musty, grainy, and herbal. The control sample was associated with smooth, soft, umami, nutty, bland, sweet, and moist attributes. The participants separated the samples based on the amount of sugar kelp addition.

**Figure 2:**
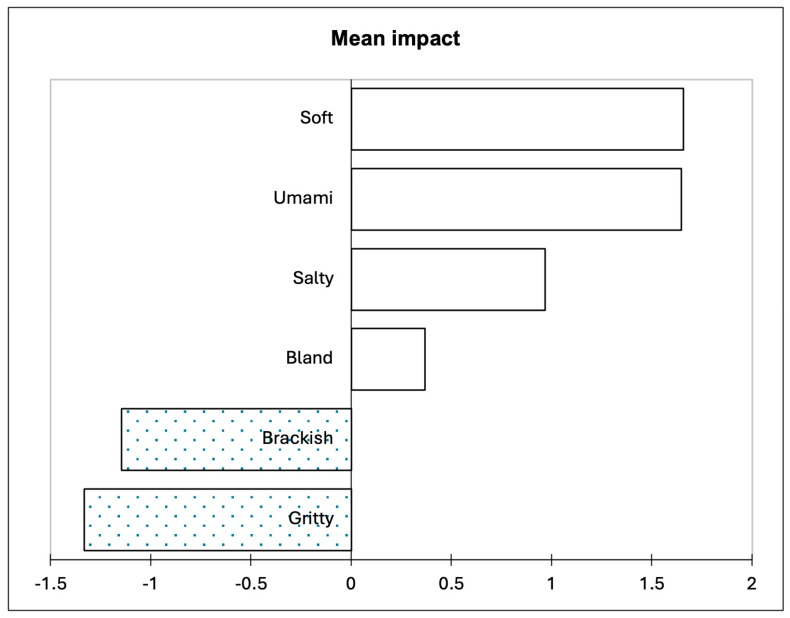
Penalty lift analysis based on the sensory terms and the overall liking of the samples. The filled bars (with dots) represent a negative impact on overall liking, and the unfilled bars represent a positive impact on overall liking. Only attributes that had a significant impact on overall liking are presented.

The overall liking scores were used in conjunction with the sensory properties from the CATA question to conduct a penalty lift analysis (Figure 2). Soft and umami had the largest positive impact on consumer liking, followed by salty and bland. Brackish and gritty were identified to negatively affect the consumers’ liking.

The consumers also identified their attitudes towards seaweed, and the mean responses are outlined in Table 2. The consumers believed that seaweed is part of a healthy diet (“Agree” on the seven-point scale) and contains many nutritional benefits (ranging from “Agree” to “Somewhat Agree”). They also identified that foods containing seaweed are expensive (“Somewhat Agree”) and that they do not usually consume food containing seaweeds (“Somewhat Disagree”). They did not believe that foods containing seaweed were disgusting (“Somewhat Disagree”). The consumers seemed interested in food containing seaweed.

### 3.2. Sensory Trial #2—Samples Evaluated with and without Nutritional Information

The consumers (n = 90) returned to the sensory lab and evaluated 6SK with and without nutritional information. Sample 6SK was selected to be included in the trial as it was not significantly different from the control in terms of overall liking as well as liking of flavour and appearance (Trial 1; Table 1). The consumers’ mean overall liking score and purchase intent for the control and the 6SK sample with and without nutritional information are outlined in Table 3. No significant differences were found between the control and the 6SK samples for both overall liking and purchase intent. Furthermore, no significant differences were found between the control and the 6SK samples’ mean overall liking scores from the first and second sensory trials.

To move beyond liking [35], the consumers were also asked to evaluate their emotional responses to the different samples (Table 4). Overall, the samples did not impact the frequency of selected emotions (21 emotions were not significantly impacted). The inclusion of nutritional information for 6SK led to a significant increase in the selection of “Happy” and “Joyful” when compared to the control. Furthermore, when the 6SK sample was presented blinded and with information, it led to an increased selection of “Pleasant” and “Enthusiastic” in comparison to the control. The presentation of the nutritional information did not significantly impact the selection of emotions, but the addition of the sugar kelp to the couscous did impact emotional response.

## 4. Discussion

The inclusion of sugar kelp impacted consumers’ acceptability of the couscous. Above the 6% level of incorporation, sugar kelp decreased the overall liking scores and the liking of the flavour. This result agrees with a past study investigating another seaweed variety, *Chlorella vulgaris,* which could be added to couscous at 6% wt/wt [21]. However, their study did not compare the seaweed-containing couscous to a control sample without seaweed. Furthermore, the results agree with past studies that identified that seaweed can be added to cereal grain-based foods, including pasta [36] and bread [37]. Fradinho and colleagues [36] identified that brown seaweed could be added to gluten-free pasta without impacting the textural characteristics. Arufe and colleagues [37] identified that *Fucus vesiculosus* seaweed powder could be added to breads at a 4% level without impacting the texture of the breads. A past study by the researchers investigating sensory perception by Nova Scotian consumers found that brown seaweed, *Ascophyllum nodosum*, and red seaweed, *Chondrus crispus*, could be added to bread and accepted at 4% and 2%, respectively [29]. Based on the results of this study, sugar kelp may be added at a higher level (6% wt/wt) to cereal grain-based products; however, the addition of sugar kelp to bread should be explored in future studies. Cereal grain-based products seem to be suitable food products for seaweed addition. The addition of sugar kelp increased the consumers’ liking of the appearance of the couscous. This result could be attributed to the fact that many vegetables and seasonings are usually mixed into couscous [38], and the consumers could have interpreted the presence of the seaweed as a seasoning or added vegetables. However, the liking scores for the appearance of 10SK were significantly lower than those of the other samples containing sugar kelp. The presence of sugar kelp in the couscous impacted the consumers’ liking of the texture, and all the samples were liked significantly less than the control. Past studies have identified that seaweed addition can impact the textural attributes and liking of pasta made with durum wheat [36,39,40].

The consumers also separated the control and the 4SK and 6SK samples from the 8SK and 10SK samples during the CATA task. As the amount of sugar kelp increased, the consumers described the samples as having a bitter and sour taste as well as being astringent, fishy, brackish, and metallic. Furthermore, the samples had an aftertaste. Fishy and brackish are well-established sensory attributes associated with seaweed [9,41,42]. Seaweed has also been perceived as bitter and sour when added to cereal grain-based products in previous studies [29,43]. Also, sugar kelp has been described as being bitter and sour as well [2,44], and a past study also identified metallic flavours in seaweeds [45]. A past study using an electronic tongue and nose identified that fermented kelp paste had an astringent aftertaste [46], and this may explain why the participants described 8SK and 10SK as astringent and having an aftertaste. The higher levels of sugar kelp also impacted the textural perception and were associated with chewy, hard, tough, crunchy, and gritty. As stated above, the addition of seaweed has impacted the textural perception of other products made from durum wheat [36,39,40]. For instance, bread made with *Palmaria palmata* had an increased hardness, agreeing with this study [47]. As identified by Quitral and colleagues [43], and in agreement with this study, as the amount of seaweed added to cereal grain products increases, the textural attributes are usually impacted negatively.

The couscous with lower amounts of sugar kelp (4SK and 6SK) was associated with salty, musty, herbal, and grainy. Herbal and saltiness have been used in past studies to describe the sensory properties of seaweed [48,49]. Musty has also been used to describe a variety of different seaweed species [50]. The couscous without sugar kelp was associated with bland, sweet, nutty, moist, umami, soft, and smooth. Soft, umami, and bland all increased consumer liking, as well as salty (Figure 2). In contrast, brackish and gritty decreased consumer liking, and both attributes were associated with the 10SK sample. Gritty has been identified by consumers to describe red seaweed added to bread [29], and as stated previously, seaweed addition has been found to impact the textural perception of cereal grain products. Grittiness is an undesirable texture in many different products that leads to consumer dislike [51]. Brackish was also used as a descriptor for sauerkraut made from sugar kelp [9]. It is interesting to note that saltiness increased liking but brackish decreased liking. Brackish is usually defined as salty water or briny. Based on the results of this study, the consumers perceived brackish to be different from salty. This result may be due to the consumers associating brackish with marine and fishy flavours, as well as being salty, as a past study found that consumers used brackish, fishy, salty, and ocean breeze to describe sauerkraut made from sugar kelp [9]. Future studies may want to ask consumers to define the terms in the CATA questionnaire to further investigate their perception of seaweeds.

Samples 4SK and 6SK were not significantly different in terms of consumer liking and were not associated with attributes that decreased liking. As such, 6SK was included in the second trial investigating consumer perception with and without nutritional information. The influence of nutritional information on consumer liking is usually product-specific [52] and is influenced by the type of claim [53]. For instance, probiotic claims on fermented milk have been found to impact consumers’ overall liking [54], but information about plant sterols on deli meat did not influence overall liking [55]. The introduction of or knowledge about nutritional information has been proposed as a method to introduce novel foods and novel ingredients [56,57]. Nutritional information about sugar kelp (a novel ingredient, as only 7% of participants identified they had previously consumed sugar kelp) did not significantly increase purchase intent or overall liking (Table 3). The result may be because consumers are not willing to compromise sensory properties for nutritional benefits [58,59,60,61]. Furthermore, front-of-pack information has been found not to influence purchase intent [62], agreeing with this study as the purchase intent was not significantly different.

As evidenced by the response to the belief questions (Table 2), the consumers agreed that seaweed has nutritional benefits. Still, the knowledge about nutritional information did not increase their liking or willingness to purchase. This reinforces that sensory properties directly influence liking and agrees with a past study on seaweed-based foods that found that taste/edibility predicts acceptability [63]. Also, familiarity influences liking [63,64], and although the participants were not regular consumers of sugar kelp, they may consume other seaweed varieties. Future studies should ask what seaweed varieties the participants regularly consume, if any. The participants in this study believed that seaweed is nutritious, and the presentation of nutritional information did not impact liking or purchase intent. A past study identified that Australian consumers found that seaweed consumption is intrinsically related to environmental sustainability [65]. The promotion of sustainability instead of nutritional benefits may increase consumers’ willingness to eat seaweed-based foods.

Emotional responses should also be evaluated when investigating novel ingredients, as it has been found that consumption behaviour and consumer choice are related to their emotions [66,67,68,69]. Emotional responses are strongly linked to sensory perception, and emotions can help determine consumers’ preference for a product [70]. Furthermore, nutritional information has been found to influence consumers’ emotional responses, which in turn impacts preference [71]. The nutritional information did not impact the selection of emotions agreeing with the results of the liking and purchase intent scores. The result agrees with a past study that identified little differences in emotional response to cheese after being presented with a health claim [72]. However, both 6SK samples (with and without nutritional information) led to an increased selection of four emotions (“Happy”, “Joyful”, “Pleasant”, and “Enthusiastic”). The emotions are all characterized as positive and have been associated with acceptability [73]. Furthermore, the emotional responses were able to discriminate between the products better than the liking scores (as no significant differences were identified; Table 3). Emotional responses have been found to be better at differentiating samples than liking scores [74,75], and that was identified in this study as well. Past studies have linked positive emotions to increased purchase intent [76], and although the purchase intent for the 6SK samples was not significantly different from that of the control, it may indicate that consumers are interested in couscous with seaweed, especially since emotional measurements have been found to improve food choice prediction [77].

The current study is not without limitations. Firstly, the consumers were recruited if they were regular consumers of couscous, but all ingredients were listed during the recruitment process so the consumers knew they would be consuming seaweed. This knowledge may have led to consumers interested in seaweed participating in our study. Also, consumer perception around the world needs to be investigated, as seaweed is considered a traditional food in Nova Scotia, Canada [78], and that may have increased consumer familiarity and, in turn, increased acceptance. Also, the promotion of the health properties of seaweed has been identified to engage consumers who are potentially interested in new experiences [79], and health consciousness has been found to influence consumer perception of seaweed-based foods [80]. Future studies should ask about willingness to try new foods and health consciousness to correlate these results with sensory perception. Furthermore, couscous is usually served as part of a meal and not by itself. If couscous was served with other food items, the consumers’ sensory perception may have differed. Lastly, sugar kelp should be ground to different particle sizes to evaluate how it impacts the sensory properties of couscous and other cereal grain-based products.

## 5. Conclusions

The development of food products containing seaweed that appeal to consumers in the Western world will add value to seaweed as well as increase consumer familiarity with it. This study found that sugar kelp can be added to couscous at 6% wt/wt without negatively impacting consumers’ liking and liking of flavour. It did impact liking of texture negatively, and future studies should investigate different drying and grinding techniques to improve textural perception. At 8% wt/wt incorporation and above, sugar kelp decreased liking and led to bitter, sour, brackish, fishy, and astringent attributes being perceived. Consumers liked couscous that was described as soft, umami, salty, and bland, i.e., couscous they did not perceive as brackish and gritty. Consumers identified that they believe seaweed is nutritious, but the presentation of nutritional information did not increase liking, purchase intent, or emotional response. This result indicates that sensory properties directly influence liking of seaweed-containing foods. Overall, the consumers liked the addition of sugar kelp to couscous up to 6%. These results should help companies incorporate sugar kelp into new food products that are acceptable to consumers in the Western world.

## Figures and Tables

**Table 1:** The mean liking scores (1 = Dislike Extremely and 9 = Like Extremely) of the couscous samples with the standard deviation in brackets (n = 99). Within columns, the samples with different letters had significantly different means at the 5% level of significance.

Sample	Appearance	Flavour	Texture	Overall Liking
Control	5.0 b (1.2)	6.6 a (1.1)	6.0 a (1.5)	6.4 a (1.6)
4SK	6.1 a (1.8)	6.7 a (1.0)	5.1 b (1.3)	6.5 a (1.6)
6SK	5.9 a (1.6)	6.6 a (1.5)	5.2 b (1.8)	6.5 a (1.7)
8SK	5.9 a (1.3)	4.9 b (1.1)	4.3 c (1.2)	4.8 b (1.4)
10SK	4.5 b (1.1)	4.9 b (1.6)	4.3 c (1.4)	4.6 b (1.3)

**Table 2:** Consumers’ beliefs about seaweed (n = 99) (the mean scores with the standard deviation in brackets). Data were input on a seven-point Likert scale, where 1 = “Strongly Disagree” and 7 = “Strongly Agree”.

Characteristic	Mean
Seaweed can be part of a healthy diet.	6.3 (1.5)
Food containing seaweed is expensive.	5.0 (1.1)
Seaweed is a good source of antioxidants.	6.0 (1.1)
Seaweed is a good source of vitamins.	5.5 (1.2)
Seaweed is a good source of omega-3 fatty acids.	6.1 (1.3)
Foods containing seaweed are disgusting.	3.5 (1.4)
Foods that contain seaweed are healthy.	5.6 (1.5)
I regularly consume products containing seaweed.	3.0 (1.7)
It is hard to find products that contain sources of seaweed.	4.0 (1.8)

**Table 3:** The mean overall liking scores (1 = Dislike Extremely and 9 = Like Extremely) and purchase intent (1 = Definitely would not purchase and 5 = Definitely would purchase) of the samples (control, 6SK Blinded, and 6SK Informed) with the standard deviation in brackets (n = 90). Within columns, the samples with different letters had significantly different means at the 5% level of significance.

Sample	Overall Liking	Purchase Intent
Control	6.1 a (1.2)	3.1 a (1.0)
6SK Blinded	6.3 a (1.2)	3.3 a (1.1)
6SK Informed	6.5 a (1.3)	3.4 a (1.1)

**Table 4:** Frequency of the emotions as selected by the consumers (n = 90) for the different samples (control, 6SK Blinded, and 6SK Informed). Post hoc multiple pairwise comparisons were performed using McNemar’s test with Bonferroni alpha adjustment. Statistically significant differences (*p* < 0.05 after Bonferroni correction) are bolded and samples with different letters, within rows, were significantly different.

Emotion	Control	6SK Blinded	6SK Informed
Happy	**26 a**	**33 ab**	**41 b**
Joyful	**17 a**	**29 ab**	**31 b**
Good	22	25	33
Interested	28	33	36
Pleasant	**23 a**	**35 b**	**40 b**
Good-natured	10	17	21
Secure	2	5	7
Satisfied	14	14	23
Free	8	9	7
Understanding	3	4	4
Enthusiastic	**6 a**	**21 b**	**19 b**
Loving	4	9	6
Adventurous	15	22	14
Aggressive	1	0	0
Wild	4	8	9
Active	10	10	9
Nostalgic	10	6	13
Warm	10	14	11
Mild	12	11	12
Calm	18	8	11
Tame	5	0	6
Bored	8	2	4
Disgusted	6	4	4
Guilty	1	2	1
Worried	3	4	2

## Data Availability

The data presented in this study are available upon request from the corresponding author [M.B.M.].

## Supplementary Materials

The following supporting information can be downloaded at https://www.mdpi.com/article/10.3390/foods13182912/s1: Table S1: Frequency of selection of sensory attributes for five different samples (n = 99).
