# Supplementary material for: Atlantic Canadians’ Sensory Perception of Couscous Made with Sugar Kelp (Saccharina latissma)
Source: Foods. 2024 Sep 14;13(18):2912. doi: 10.3390/foods13182912 (PMC11431538; doi:10.3390/foods13182912)
Supplement: Supplementary file 1 [file foods-13-02912-s001.zip › foods-3121016-supplementary.pdf]

Supplementary Table S1. Frequency of selection of the sensory attributes for the five different samples (n=99)

Attributes	Control	4SK	6SK	8SK	10SK
Umami***	68a	53bc	68a	38c	26c
Crunchy**	17b	12b	22aab	17ab	32a
Moist**	24a	10ab	13ab	4b	4b
Herbal**	4a	9ab	5a	4a	13b
Metallic**	16a	22ab	22ab	32b	33b
Fishy**	4a	7a	7a	10ab	16b
Sweet^{ns}	24	24	24	17	18
Bitter**	8a	15ab	27b	21ab	25b
Astringent***	2a	5a	8a	19b	19b
Salty^{ns}	58	68	59	58	59
Nutty^{ns}	10	8	5	10	7
Grainy^{ns}	9	11	9	9	6
Smooth*	19a	19a	10ab	6b	6b
Chewy^{ns}	3	4	4	3	9
Sour^{ns}	4	4	7	7	12
Hard^{ns}	8	7	5	8	10
Aftertaste***	8a	18ab	23bc	32bc	42c
Bland***	56a	46ab	34bc	29bc	22c
Fatty^{ns}	22	18	11	20	23
Tough^{ns}	4	4	5	8	8
Gritty***	18a	25a	25a	29ab	35b
Brackish***	12a	27b	32b	36bc	42c
Musty*	7a	8a	12ab	18b	18b
Soft***	47a	32b	27b	26b	24b

*** Indicates significant differences between samples according to Cochran's Q test at $p < 0.0001$.
** Indicates significant differences between samples according to Cochran's Q test at $p < 0.01$.
* Indicates significant differences between samples according to Cochran's Q test at $p < 0.05$.
ns Indicates no significant differences between samples according to Cochran's Q test ($p < 0.05$).
[1] Post hoc multiple pairwise comparisons were performed using McNemar's test with Bonferroni alpha adjustment. The different letters (a, b, c) denote significant significance differences within the attribute at $p < 0.05$.
